# Autonomous dynamic obstacle avoidance for bacteria-powered microrobots (BPMs) with modified vector field histogram

**DOI:** 10.1371/journal.pone.0185744

**Published:** 2017-10-11

**Authors:** Hoyeon Kim, U. Kei Cheang, Min Jun Kim

**Affiliations:** 1 Department of Mechanical Engineering, Southern Methodist University, Dallas, TX, United Stated of America; 2 Department of Mechanical Engineering and Energy Engineering, South University of Science and Technology, Shenzhen, Guangdong, China; Chongqing University, CHINA

## Abstract

In order to broaden the use of microrobots in practical fields, autonomous control algorithms such as obstacle avoidance must be further developed. However, most previous studies of microrobots used manual motion control to navigate past tight spaces and obstacles while very few studies demonstrated the use of autonomous motion. In this paper, we demonstrated a dynamic obstacle avoidance algorithm for bacteria-powered microrobots (BPMs) using electric field in fluidic environments. A BPM consists of an artificial body, which is made of SU-8, and a high dense layer of harnessed bacteria. BPMs can be controlled using externally applied electric fields due to the electrokinetic property of bacteria. For developing dynamic obstacle avoidance for BPMs, a kinematic model of BPMs was utilized to prevent collision and a finite element model was used to characteristic the deformation of an electric field near the obstacle walls. In order to avoid fast moving obstacles, we modified our previously static obstacle avoidance approach using a modified vector field histogram (VFH) method. To validate the advanced algorithm in experiments, magnetically controlled moving obstacles were used to intercept the BPMs as the BPMs move from the initial position to final position. The algorithm was able to successfully guide the BPMs to reach their respective goal positions while avoiding the dynamic obstacles.

## Introduction

It is well established that microscopic scale robotics has a high potential to be utilized in biological, medical, and industrial applications; despite facing many challenges. For biomedical engineering, core tasks such as localized/targeted drug delivery, micro invasive surgery, cell manipulation, biosensing, cell sorting, and cell fusion can be performed [1–6]. In the field of industrial engineering, microrobots have shown their capabilities to complete microscale tasks such as micro-assembly, transport, precision micro-machining, and micro-manipulation [7–9]. In order to develop swimming microrobots for these applications, there have been various challenges. The major challenge is to propel microrobots at a low Reynolds number fluidic environment where the conventional macro scale swimming methods do not work due to the negligence of inertia force caused by relatively large viscous forces. Therefore, the generation of a propulsive force to power microrobots has become a primary problem to be addressed. The widely discussed solution for low Reynolds number propulsion is to generate nonreciprocal motion which can be achieved using corkscrew motion or deformable bodies [10] that are inspired by the movement of microorganisms and structures formed in nature [2, 11–14]. One of the previously explored designs is the artificial helical shaped microrobot that is coated with a metal and manipulated by magnetic fields [15, 16]. In addition, self-propelled micromotors have been studied using spherical Janus particles within the last decade [17].

Another challenge is the autonomous control of microrobots and the interest of this challenge becomes significantly increasing. A navigation system will play an important role in microrobotics to complete the aforementioned tasks due to reducing any damage to the microrobot and minimizing adverse effects. In addition, the autonomous control method can supply highly efficient navigation when compared to manual motion control. Hence, the development of advanced motion control techniques is necessary for microrobots to achieve their tasks. For this purpose, motion modeling of a microrobot has been built for control and validated by manual control inputs in experiments [7, 18]. Furthermore, both biological and non-biological microrobots have been navigated using autonomous path planning [19–21]. The path planning method ensures that the microrobot arrives at the goal position. However, in a real environment, there will be not only static but also dynamic obstacles; hence, dynamic obstacles should be considered for autonomous motion control. Herein, we suggest a dynamic obstacle avoidance algorithm for BPMs.

In this paper, an autonomous motion control algorithm was developed for avoiding dynamic obstacle in real-time. We designed the experimental setup to test the algorithm using magnetically controlled moving obstacles. Then, we demonstrate the feasibility of our approach experimentally by using the moving obstacles to intercept the paths of BPMs. We manufactured microrobots which use biomolecular motors from bacteria. Using the galvanotaxis of the bacteria, these microrobots can be driven by applied electric field. The autonomous navigation algorithm for static obstacle avoidance was developed [22] after the controllability and mobility of BPMs were demonstrated in previous works [2, 23]. For this work, a dynamic obstacle avoidance algorithm is capable of computing the optimal control inputs to avoid dynamic obstacles in real-time and allows the guidance of a BPM to reach the target position without using path planning. This capability will allow BPMs to adapt to changeable environments and greatly increase their versatility for microscale applications.

To develop a dynamic obstacle avoidance algorithm, several factors need to be considered due to the characteristics of the BPMs’ biological actuators and the design of the control system. The first factor is an inherent motion caused by a densely packed mono layer of bacteria, called bacterial carpet. This motion induces uncontrollable movements which lead to high probability of collision. However, the studied kinematic model of BPMs can be used to predict this motion and help to plan a safe motion. Another factor is a deformation of the electric field around dynamic obstacles. The presence of dynamic obstacles leads to non-uniform electric fields which can cause BPMs to move in undesired directions. In our proposed method, all these constraints are taken into account and the potential risk caused by dynamic obstacles is prevented in real-time. Our proposed algorithm was based on the simulation results in [24] and we focused on proving the reliability of the algorithm using magnetically controlled moving obstacles. Furthermore, the performances of the experimental results are quantified by the danger index representing the potential risk. The defined danger index was calculated using the control input and the relative position between a BPM and surrounding obstacles.

## Materials and methods

### Bacteria-powered microrobots

#### Configuration of bacteria-powered microrobots

**Fig 1:**
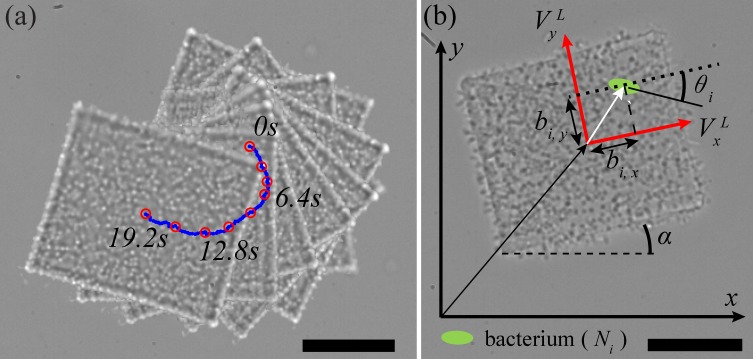
Introduction of BPMs. (a)Self-actuation induced by bacterial carpet during 19.2s, (b)A Schematic of the BPM (ellipse: bacterium, Ni: *i*-th bacterium).The scale bar represents 20 μm.

A BPM is an integrated robotic system using the bacteria called *Serratia marcescens* and an inorganic material called SU-8. Using a slimy material secreted from the bacterial bodies, these bacteria can be attached onto the surfaces of SU-8 microstructures [25, 26]. The SU-8 Structures, which serve as the bodies of the BPMs, are fabricated using photolithography. Through blotting directly on a bacterial colony on an agar plate, hundreds or thousands of bacteria can be harnessed onto the surface of the microfabricated SU-8 structures [27]. The blotted structures are released in water based fluids through the use of a water-soluble sacrificial layer [28]. These released microstructures, BPMs, are free to move through the fluid, as shown in Fig 1A.

#### Propulsion of BPMs

To generate propulsive force at low Reynolds number, the BPMs utilize hydrodynamics of flagella from the bacterial carpet attached to the bottom of the SU-8 microstructure. The hydrodynamics is the result of individual bacteria flagellar waving [29]. The flagella on the carpet undergo corkscrew motions which are nonreciprocal and the collective motion of the flagella helps the BPM to overcome friction on the bottom surface and the viscosity present in a low Reynolds fluid. Moreover, the harnessed bacterial propulsion generates self-actuated motion without external stimulus. Fig 1A represents the self-actuation of a BPM during 19.2s with a clockwise rotation. The self-actuation will be useful to adjust the orientation of the BPM as examined in [23]. The negatively charged bacteria bodies will generate electrophoretic motion in the presence of an electric field. By controlling the direction and the magnitude of the electric field, we were able to generate translation motion of BPMs.

#### Stochastic model for BPMs

There are two velocity components for translational movement which depends on *x*-axis and *y*-axis in the local coordinate frame with respect to the center of mass [14]. For the rotational motion of the BPM, the angular velocity, $\dot{\alpha }$, is related to the position of the *i-*th bacterium and its orientation angle *θ*_*i*_ as shown in Fig 1B. As a result, the equations of translational and rotational velocity of self-actuation can be expressed by

$$\left[\begin{array}{c} V_{x}^{L}\\ V_{y}^{L}\\ \dot{\alpha } \end{array}\right]≔\bar{p}\left[\begin{array}{c} \frac{1}{k_{T}}\sum_{i=1}^{N_{b}}\cos\theta_{i}\\ \frac{1}{k_{T}}\sum_{i=1}^{N_{b}}\sin\theta_{i}\\ \frac{1}{k_{R}}\sum_{i=1}^{N_{b}}\left(b_{i,x}\sin\theta_{i}-b_{i,y}\cos\theta_{i}\right) \end{array}\right]=\bar{p}\left[\begin{array}{c} \beta_{1}\\ \beta_{2}\\ \beta_{3} \end{array}\right]$$ (1)

where *N*_*b*_ and *θ*_*i*_ are the number of bacteria and the orientation of bacteria on the surface of the microstructure, and *k*_*T*_ and *k*_*R*_ are the translational viscous drag coefficient and rotational viscous drag coefficients, respectively. The inherent movement of BPMs is dependent upon parameters *β*_*1*,*2*,*3*_ and $\bar{p}$. The values for *β*_*1*,*2*,*3*_are determined from the number of adhered bacteria, the distribution of the attached bacteria, and their respective orientation *θ*_*i*_ [23, 30]. Furthermore, the propulsive force from each bacterium affects the resultant motion. The average of the propulsive force $\bar{p}$ is 0.45 pN [31].

As a result of previous work [23], the BPM’s movement with a control input can be described by combining the kinematic model with electrokinetic actuation on a global coordinate system, as follows:

$$\left[\begin{array}{c} V_{x}\\ V_{y}\\ \dot{\alpha } \end{array}\right]=R_{z}(\alpha_{t_{i-1}})\left[\begin{array}{c} V_{x}^{L}\\ V_{y}^{L}\\ \dot{\alpha } \end{array}\right]+∆d(U_{x},U_{y})/t_{s}=\bar{p}\left[\begin{array}{cc} R(\alpha_{t_{i-1}}) & \begin{array}{c} 0\\ 0 \end{array}\\ \begin{array}{cc} 0 & 0 \end{array} & 1 \end{array}\right]\left[\begin{array}{c} \beta_{1}\\ \beta_{2}\\ \beta_{3} \end{array}\right]+\left(\begin{array}{c} U_{x}\\ U_{y}\\ 0 \end{array}\right)\beta_{4}/t_{s}$$ (2)

where *R*_*z*_(*θ*) is a rotation matrix about *z*-axis and *Δd*(*U*_*x*_, *U*_*y*_)/*t*_*s*_ is the velocity by electokinetic actuation. Herein, we define *U*_*x*_ and *U*_*y*_ are the input voltages for the coordinating mobility, *t*_*s*_ is the sampling time. *β*_*4*_ represents the electrophoretic property from the total charge of the attached bacteria.

The motion of the BPM can be represented by parameters *β*_*1*,*2*,*3*_, and *β*_*4*_ when the control inputs are presented. In addition, the expected locomotion of a BPM can be calculated with the control input voltages which helps to evaluate the collision risk using this stochastic model.

#### Validation of a BPM model

The stochastic model, as indicated in (2), was validated by simulating the motion after obtaining the necessary parameters *β*_*1*,*2*,*3*_ from the experimental data. For this validation, a 40 × 43 μm^2^ rectangular structure was used in the real experiment and the control input was 20 V/cm on right (+) direction on *x*-axis.

In order to conduct the simulation, the parameters were obtained by using two motion videos; one is self-actuation motion and the other is electrokinetic motion with 10 V/cm. The position vectors that include Cartesian position and the BPM orientation were utilized after image processing of experiment video. First, the parameter *β*_*3*_ was extracted by calculating the orientation of the BPM without any input, and the value of *β*_*3*_ is 0.38 ± 0.07 rad/(s·pN). Next, the *β*_*1*_ and *β*_*2*_ were calculated while excluding the control input matrix in (2) and were 2.68 ± 3.71 μm/(s·pN) and -3.57 ± 5.28 μm/(s·pN), respectively. Finally, we could get the *β*_*4*_ by substituting the constant control input *U*_*x*_ (10V/cm) in (2), which was 0.17 ± 0.03 μm/(V/cm).

**Fig 2:**
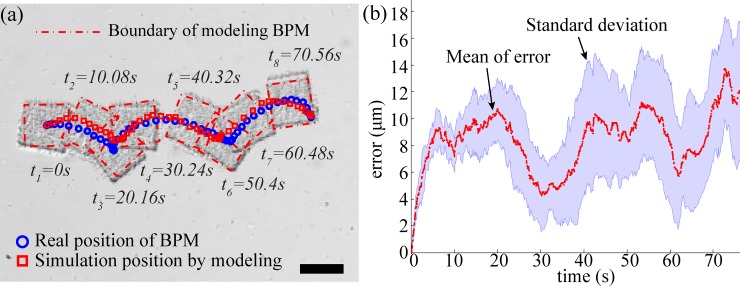
Comparison between the real motion of the BPM and the simulation result of the stochastic model. (a)Position of real experimental data and modelling data, (b)Locomotion error between experimental result and simulation result. The scale bar represents 40 μm.

In Fig 2, the simulation result was conducted by the normal distribution of each parameter and compared with the real experimental positions of the BPM with 20 V/cm. The simulated BPM began with the same position as the experimental position. The simulated motion closely corresponded to the experimental consecutive images as shown in Fig 2A with 8.59 ± 1.27 μm errors for the position between simulation and real data (Fig 2B). The error fluctuations between experimental and simulation results are due to the unpredictable behavior of bacteria. In simulation, the kinematic parameters were constant and the accumulated mismatched position between simulations and experiments led to a growth in the standard deviation of error. This result is one of examples; other examples are indicated in previous work [14, 23, 24]. Hence, the kinematic model of the BPMs is validated for developing an obstacle avoidance algorithm in terms of predicting the BPM’s locomotion and calculating the collision risk.

### Dynamic obstacle avoidance strategy

In our previous work [22], the static obstacle avoidance approach based on the dynamic window approach (DWA) [32] was well demonstrated in various environments. In the case of moving obstacles, however, the unpredicted motion of dynamic obstacles can increase the probability of collision. To strengthen the obstacle avoidance algorithm under moving obstacles, the vector field histogram (VFH) method [33] was added in our proposed method.

### Considering elements for motion control of BPMs

The natural self-actuated motion of BPMs and the deformation of electric field around obstacles are the two main factors to consider when developing the dynamic obstacle avoidance algorithm. The primary concern is related to a bacterial carpet that exhibits self-actuation of a BPM which is naturally induced by flagellar motors. If the control input is not determined by considering the self-actuation of a BPM, there will be a high probability of collision with obstacles. Moreover, the velocity of dynamic obstacles can move relatively fast comparing BPMs and the sudden movement of dynamic obstacles will cause high risk situation. The other consideration is the deformation of an electric field around the obstacle area creating non-uniformities in the potential field. This deformation can be understood by the analog of a river flow around rock where the water flow around the rock is nonlinear and non-uniform. Similarly, the electric field will be distorted around a moving obstacle. This field deformation can lead to undesired motion of the BMP which increases the probability of collision with the moving obstacles. The phenomenon of non-uniform potential field was simulated using a finite element program called COMSOL Multiphysics. In the simulation, the dynamic obstacle was composed of nickel which allow for magnetic actuation and non-permeability as boundary conditions.

**Fig 3:**
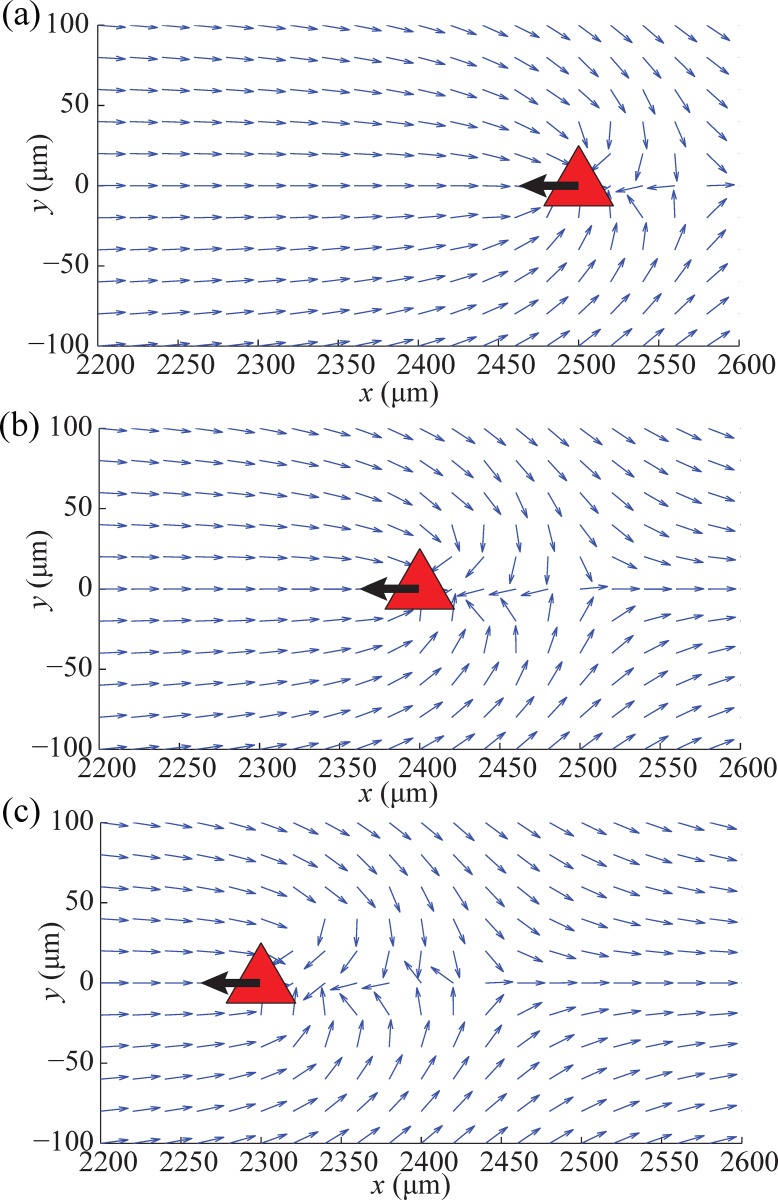
COMSOL Multiphysics simulation results of electric field flows. (a)Electric potential flow at 0s, (b)10s, and (c)20s. The triangle represents a moving obstacle that has a nickel material.

The simulation results indicate the distorted range around the moving obstacle over time, as shown in Fig 3. A 10 V/cm electric field was applied in the +*x*-direction. The triangle object represents a dynamic obstacle with a velocity of 10 μm/s toward the left side from the initial position at 0s (Fig 3). The arrows of electric field deform around the obstacle at 20s (Fig 3C).

**Fig 4:**
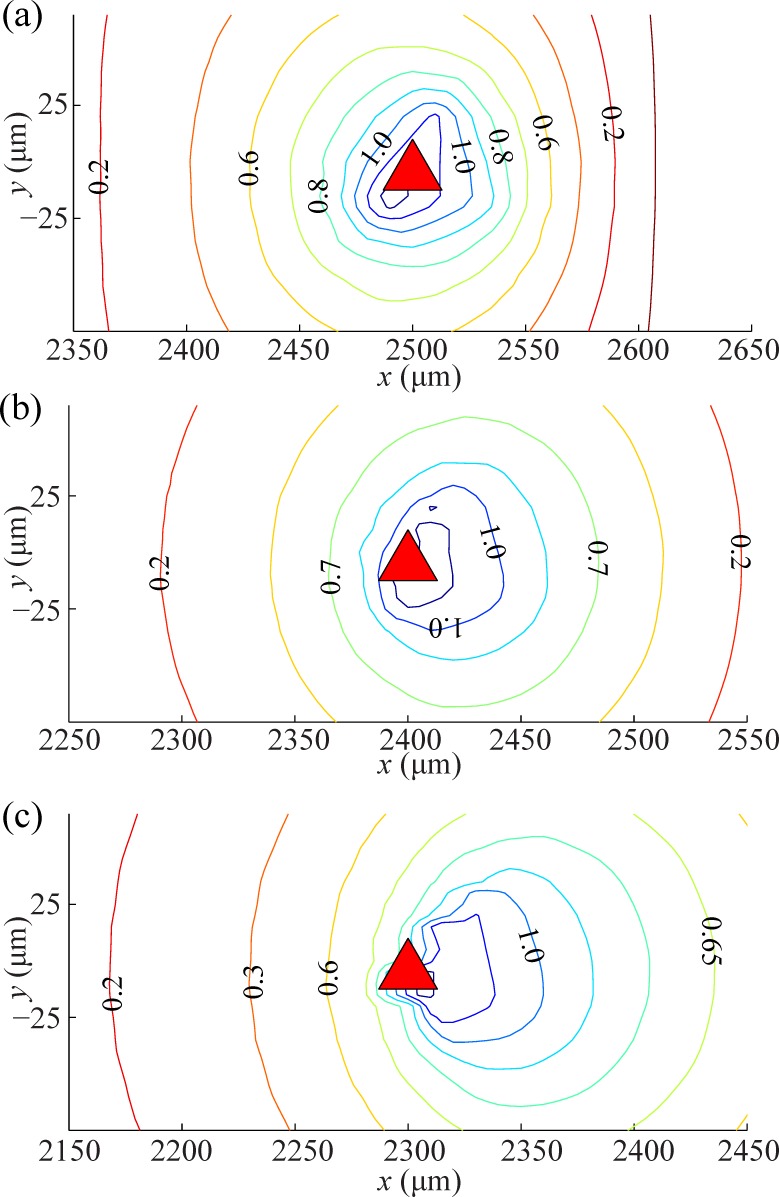
COMSOL Multiphysics simulation results of distorted electric field area. (a)Non-uniformity of potential field at 0s, (b)10s, and (c)20s. The triangle represents a moving obstacle and the contour values indicate a quantity of distortion for electric field.

The distorted electric field was quantified using the different angle between the desire direction and the direction of electric field. In Fig 4, the streamline indicate the quantity which is normalized from 0 to 1. The larger the value is, the larger the deformation becomes. The non-uniform electric potential region forms ripples which disperse from the dynamic obstacle. Using this profile of the electric field, the formation of distorted area can be utilized in the dynamic obstacle avoidance algorithm to ensure a BPM to avoid distorted regions.

#### Suggested approach for dynamic obstacle avoidance

Our proposed approach for the development of dynamic obstacle avoidance of BPMs is based on the integration of modified DWA and redefined VFH. We defined four different objective functions in a main function (DWA) to select an optimal control input by considering the parameters listed above, as the BPM approaches a goal position. However, in dynamic environments where moving obstacles are presented, the objective function would not be enough to consider the motion of the dynamic obstacles because the objective function only considers the surrounding environment at a given specific moment. Instead of adding other functions to the objective function, the role of the redefined VFH is to filter out the control inputs with high risk in advance by considering the distance between the BPM and the dynamic obstacles.

The search for control inputs is carried out directly in the space of voltages with–π to +π range of the direction for electric field. In the suggested algorithm, the search space of control inputs depends on the maximum input voltage in the system setup.

**Fig 5:**
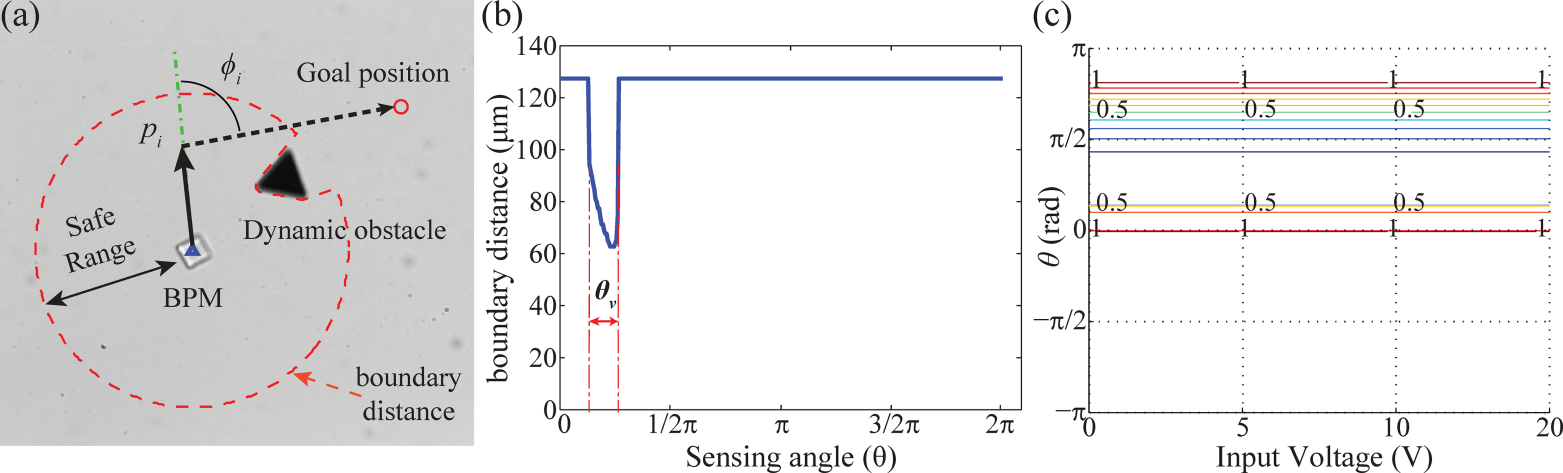
Processing to extract risk control inputs using modified VFH. (a)BPM situated in environment with an obstacle, the *p*_*i*_ is the position by input (*U*_*i*_, *θ*_*i*_), (b)The vector histogram of boundary distance within safe range in (a), (c)Contour graph of the VFH function cost.

There are three steps in the suggested algorithm to determine the commands controlling the BPM. In the first step of the algorithm, the modified VFH function is implemented to restrict the candidate control inputs from the search space when obstacles are located within the safe range as shown in Fig 5A. Even though the distance between the BPM and the obstacle was far initially, if the dynamic obstacle moves close to the BPM at high speed, the BPM might not be able to avoid collision in short time intervals. Therefore, we used the concept of VFH to exclude the motion inputs that drive a BPM toward the valley, occupied by the dynamic obstacles, in vector histogram. The inputs heading for obstacles will have 0 value from the defined VFH function (ν(*U*, *θ*)) as follow:

$$\theta_{v}=\{\theta_{i}\}\;for\;BD(\theta_{i})\leq safe\;range,\;1\leq i\leq 360$$ (3)

$$\begin{array}{l} \;T_{\theta }=\min\left(\left|\theta -\theta_{v}\right|\right),\\ v(U,\theta )=\left\{\begin{array}{cc} 0 & if\;T_{\theta }\leq \varepsilon_{s}\\ \frac{T_{\theta }^{2}}{T_{max}^{2}} & if\;\varepsilon_{s}<T_{\theta }\leq T_{max}\\ 1 & if\;T_{max}\leq T_{\theta } \end{array}\right\} \end{array}$$ (4)

where ***θ***_***v***_ is the group of valley angles in vector histogram, as shown in Fig 5B, *T*_*θ*_ is the minimum gap between the direction of a control input and the angles ***θ***_***v***_, Function *ν*(*U*, *θ*) represents the distance of the BPM from the valley, and ε_s_ is the tolerance used to extend the valley range due to self-actuation. For instance, the angle *θ* from 23° to 74° has a value of 0 (Fig 5C), thus, these control inputs will be excluded when the VFH is combined with the main objective function.

In the second step, the objective function will be applied to compute the main function (DWA). In the main function (DWA), there are four objective functions: *heading*, *movement*, *clearance*, and *control* which stand for the quantity value of individual performance (motion direction toward a goal, long movement motion, collision risk, following controllability in sequence). It is given by:

f(U,θ)=γ∙heading(U,θ)+δ∙movement(U,θ)+ω∙clearance(U,θ)+σ∙control(U,θ) (5)

where the *U* and *θ* are a magnitude of an admissible voltage and a desired direction of electric field. The function of *f* is the weighted sum of four components with the weights of *γ*, *δ*, *ω*, and *σ*.

The concerns mentioned in previous section are accounted for in the *clearance* and *control* function. In *clearance* function, the collision risk from motion of a BPM can be calculated using the kinematic model (2) with control inputs *U*_*x*_, *U*_*y*_, (*U*_*x*_ = *U·*cos*θ*, *U*_*y*_ = *U·*sin*θ*). The *control* function tends to pick up the control input that enables a BPM to be located at the controllable region (non-distorted regions) where the direction of the generated electric field matches with the direction of the control input. The objective function can lead a BPM to a goal position using the *heading* function since it enables a BPM to steer towards the goal. To make a BPM move a longer distance, we include the *movement* function in the objective function.

The sum of each function in (5) will be computed with respect to the position of a BPM, the goal position, and boundary distance information, as shown in Fig 5A. The *heading* function is to find the most favorable input direction which has the smallest *ϕ*_*i*_ by calculating the angle at *p*_*i*_ in Fig 5A as follows,

$$heading(U_{i},\theta_{i})=1-\frac{\phi_{i}}{\pi }$$ (6)

**Fig 6:**
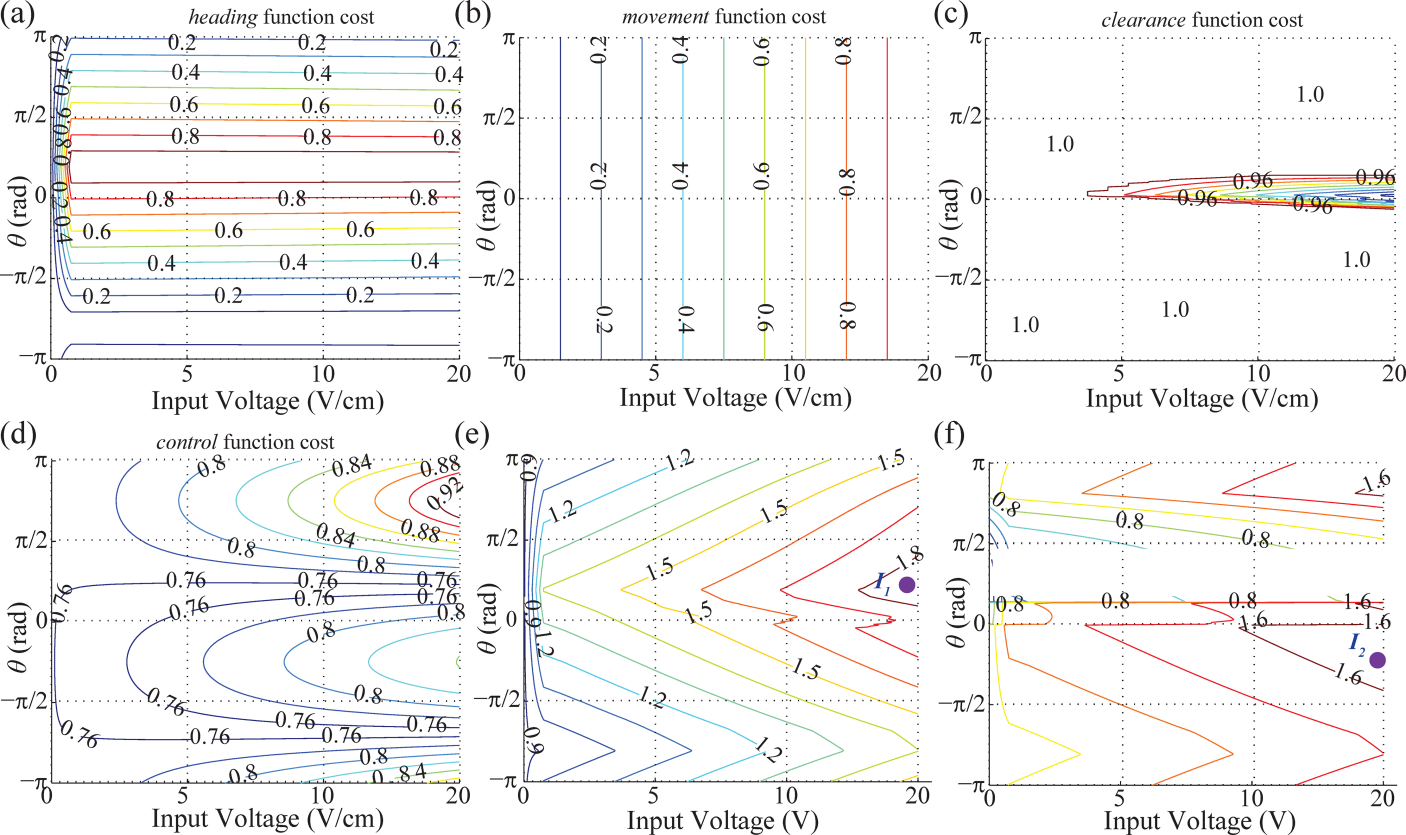
Contour graph for calculation for final control input. (a)Result of *heading* function, (b)Result of *movement* function, (c)Result of *clearanc*e function, (d)Result of *control* function, (e)Result of the objective function by the sum of all functions from (a-d) before combining VFH cost (Fig 5C), (*I*_*1*_:the chosen control input), (f)Recalculated result after combining VFH cost to (e)(*I*_*2*_: the recalculated control input after combining VFH).

A small *ϕ* means that a BPM is closely aligned to the direct course to the goal. The computed cost is shown in Fig 6A. The *θ* angle inputs (ranged from 0° to 90°) have the highest cost when the goal is located as shown in Fig 5A. The cost of movement function is given as,

$$movement(U_{i},\theta_{i})=\beta_{4}t_{s}\sqrt{U_{x}^{2}+U_{y}^{2}}/dist_{max}$$ (7)

where *dist*_*max*_ = *β*_*4*_*U*_*max*_$\sqrt{2}$ is the maximum movement from a maximum input voltage *U*_*max*_ on both axes for one interval. For the *movement* function, higher voltages have higher costs regardless of *θ*, as indicated in Fig 6B, because the displacement of a BPM is proportional to the magnitude of an input voltage.

Comparing with previous work [21, 22], the *C*-space is not suitable to check the collision in *clearance* function due to mobility of obstacles and computation time for the dynamic obstacle avoidance algorithm. Herein, the boundary distance (BD) from the center of a BPM is used to evaluate the potential risk of collision with obstacles. The instant boundary distance makes it possible to calculate the closest distance between the next expected position of a BPM under the control input and obstacles. The cost of *clearance* is 1.0 in the collision free case and 0 for a collision as explained in Fig 6C. For the rest of the cases, the cost of *clearance* is computed as follows,

$$clearance(U_{i},\theta_{i})=1-\frac{BD(\theta_{i})-\beta_{4}U_{i}t_{s}}{safe\;range}$$ (8)

In case of the *control* function, it is effective to adapt the gradient profile characterized around an obstacle obtained from the COMSOL Multiphysics simulation (Fig 4). Thus, the *control* function computes the controllability of a BPM at the determined position by the control inputs (*U*, *θ*) as follows,

$$control(U_{i},\theta_{i})=1-\frac{\sum \deg\left(p_{i},obstacle\right)}{2\pi }$$ (9)

where the deg function computes how large area has non-uniform electric field gradient from the predicted position of BPM by dynamic obstacles. If the estimated position by the control inputs is near the outside of the distorted gradient boundary, the *control* function has higher cost. The total resultant cost is depicted in Fig 6D.

After the objective function is computed as shown in Fig 6E, the last step is to find the optimal control input using

$$Input(U,\theta )=max(\{f(U_{i},\theta_{i})∙v(U_{i},\theta_{i})\;:\;1\leq i\leq M\})$$ (10)

where *M* is the total number of candidate control inputs. By combining the main objective function *f(U*, *θ)* with the function ν(*U*, *θ*), our approach can choose the control input which enables a BPM to avoid the approaching obstacles in advance. For instance, the control input *I*_*1*_ in Fig 6E is chosen as a result of using only the objective function. Under this input, the BPM moves toward the obstacle because there is a distance between the BPM and the obstacle. However, if the obstacle is moving fast towards the BMP, there will be a high probability for the BPM to collide with the obstacle under the control input *I*_*I*_. On the other hand, the proposed method excludes the occupied input angles that has 0 value in ν(*U*, *θ*). As a result, the control input *I*_*2*_ is selected by choosing the highest peak value in Fig 6C. The resultant performance can be different depending on tuning the weighting parameters *γ*, *δ*, *ω*, and *σ* in (5). In Fig 6A and 6C, the weight values are equal to 0.5.

As a summary, the integrated function will choose the safe control input among the available inputs using the BD data at the instant position of a BPM through implementation of the algorithm in real- time and the BPM moves toward the goal position.

#### Danger index for evaluation of performance

In order to review the performance of the proposed method in different conditions, the danger index was used as an appropriate criterion to evaluate the potential collision risk by the chosen inputs. The danger index is one of general quantitative methods in robotics for safety strategy. To quantify the danger index, there are two elements to evaluate the potential collision risk with obstacles. The first factor is the relative distance between a BPM and obstacles. The distance with obstacles is important to prevent collision events. The BPM can avoid collision as long as the BPM maintains a sufficient distance with obstacles. Thus, we can use the distance information from experiment results to calculate the criteria

$$g_{D}(D_{BO})=\left\{\begin{array}{cc} 0 & if\;D_{BO}\geq D_{max}\\ k\left(\frac{1}{D_{BO}}-\frac{1}{D_{max}}\right) & if\;D_{BO}<D_{max} \end{array}\right\}$$ (11)

$$where\;k=\frac{D_{max}}{D_{min}},\;D_{min}=R_{BPM}*kv+d_{self},\;kv=\frac{{\bar{V}}_{obs}}{{\bar{V}}_{BPM}}$$

where *D*_*BO*_ is the distance between the BPM and the obstacle, *D*_*min*_ is the minimum allowable distance between the BPM and the obstacle. The *D*_*min*_ was calculated using the radius of a BPM and the displacement of self-actuated BPM. The parameter *kv* represents the ratio of average velocity of an obstacle to the average velocity of a BPM.

Although the given control input enables a BPM to keep away from obstacles, the moving obstacle can intercept and move close to the BPM during experiments. In such a case, however, if the motion control can steer the BPM to escape the area, the collision risk will decrease. Therefore, we regarded the relative angle of the control input as the second factor. The potential risk of the second factor can be evaluated as followed

$$g_{A}(A_{CO})=\left\{\begin{array}{cc} 0 & if\;A_{CO}\geq A_{block}/2+∆\alpha \\ \frac{A_{CO}}{A_{block}(D_{BO})/2+∆\alpha } & if\;A_{CO}<A_{block}/2+∆\alpha \end{array}\right\}$$ (12)

where *A*_*CO*_ is the gap of the angle between the control input direction and the heading angle from the BPM to the center of an obstacle, *A*_*block*_ is the blocking angle between an obstacle and the center of the BPM. Therefore, if the control input steers a BPM away from the obstacle, the collision risk will become small.

The product-based danger index is then computed as a product of these contributing factors that are scaled:

$$danger\;index=g_{D}(D_{BO})∙g_{A}(A_{CO})$$ (13)

The danger index is indicated within the range 0 to 1. A high danger index occurs when both of the distant factor and the relative angle factor have high potential risk. After the data were obtained from experiment results, the danger index was computed with respect to time.

### Experimental setup

The algorithm was demonstrated with artificial dynamic obstacles that were manually controlled by a user in experiments. The dynamic obstacles were coated with nickel; thus, they were controllable using magnetic fields. Our experimental system consists of a vision system to track a target BPM and to recognize an environment in real-time.

#### Preparation of BPMs & dynamic obstacles

To create BPMs, *S*. *marcescens* were attached on the surface of the microfabricated structures with the dimensions of 25 × 20 μm^2^ and 3 μm thickness. The patterned microstructures were made of SU-8 and there was a dextran sacrificial layer under the structures to release BPMs in the fluids after blotting. More detailed information is in [22] (see S5 File also).

For creating moving obstacles, there were two main steps to fabricate triangular dynamic obstacles. The first step was to use the standard photolithography to make SU-8 bodies of dynamic obstacles. We used chrome mask with equilateral triangle patterns with the length of 43 μm for each side. The triangular dynamic obstacles have a thickness of 3 μm and can be released using a dextran layer, same as the BMPs. To enable magnetic control, Nickel pellets (99.995%, Kurt J.Lesker, PA, USA) were deposited on the structures using chemical vapor deposition at a chamber with a rate of 0.1–0.5 Å/s and pressure of 10^−7^ torr.

After depositing a 200 nm nickel film on the top of structures, they were magnetized by placing the structures overnight underneath a permanent neodymium-iron-boron magnet (K&J Magnetics, Pipesville, PA) which has surface field strength of 160.1mT. Before we used the dynamic obstacles in experiment, the magnetized nickel coated obstacles were released in a petri dish that was filled with motility buffer (0.01 M potassium phosphate, 0.067 M sodium chloride, 10^−4^ M ethylendediaminetetraacetic acid (EDTA), 0.01 M glucose, pH 7.4). Then, the undamaged magnetic dynamic obstacles were transferred, via micro pipetting, from the petri dish to the experimental chamber where experiments were performed.

#### System design for experiment

**Fig 7:**
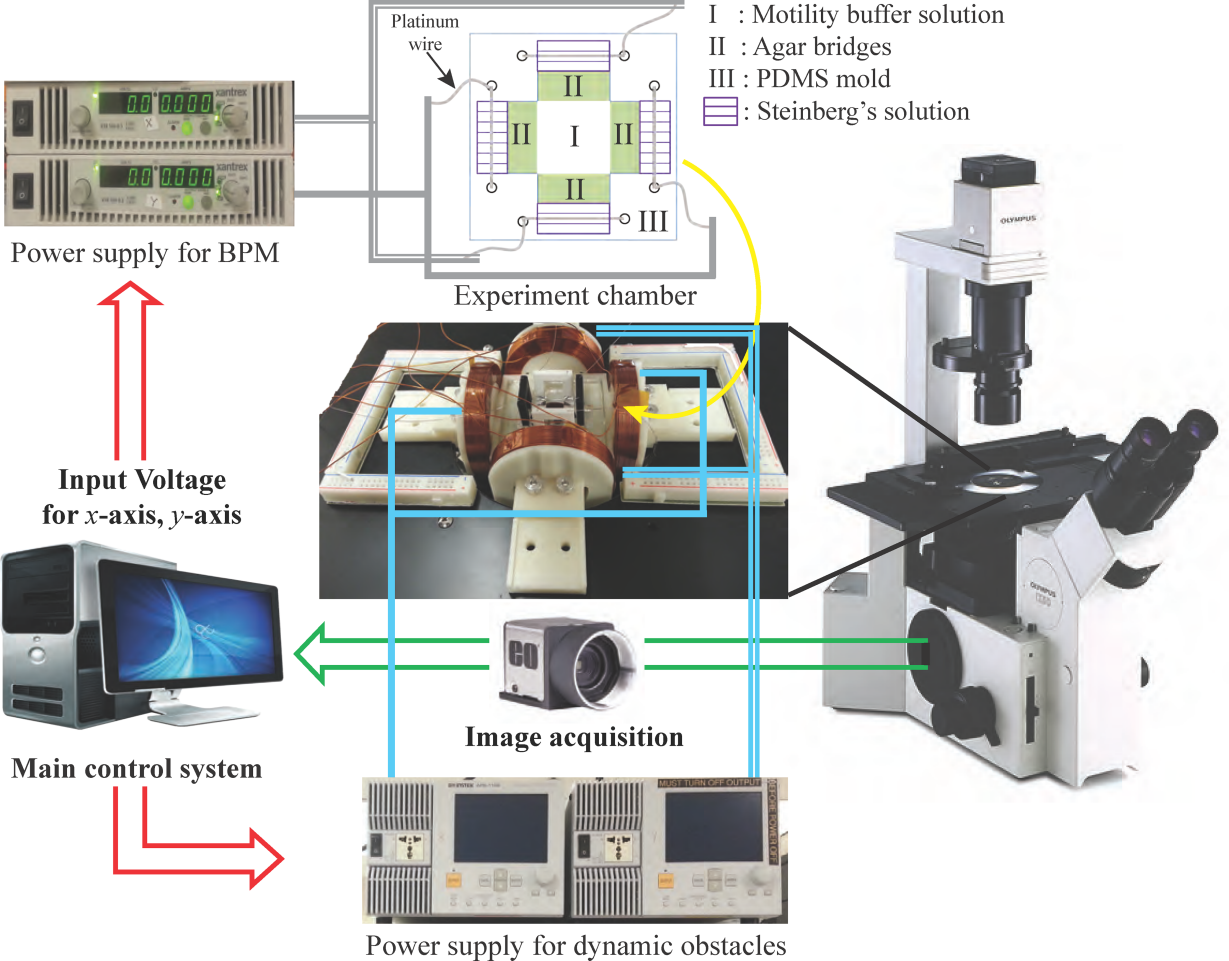
Experimental equipment and control strategy.

The experimental system setup is composed of a CMOS camera installed on an inverted microscope (Olympus IX50), an electrokinetic chamber, an electromagnetic coil stage, two power supplies (Ametek XTR 100–8.5) for a BPM, two power supplies (GW INSTEK APS-1102) for dynamic obstacles, an extra power supply for *z*-coil on stage, and DAQ board (NI DAQ SCB68). Fig 7 shows the experimental setup.

The electrokinetic chamber is the main workspace where a BPM and nickel coated obstacles were placed. The chamber was located in the center of the electromagnetic coil stage. The electromagnetic coil stage consists of two pairs of electromagnetic coils and connected to two power supplies to generate magnetic fields. To detach the non-movable dynamic obstacles from the bottom surface due to the weight and friction between the glass substrate and SU-8 bodies, the *z*-coil was applied to generate lift force.

In the experimental chamber, direct current electric fields were generated via agar salt bridges, Steinberg’s solution (60 mM NaCl, 0.7 mM KCl, 0.8 mM MgSO_4_·7H_2_O, 0.3 mM CaNO_3_·4H_2_O) and platinum wires (Fig 7). The agar salt bridges were installed to prevent contamination of possible electrode byproducts. Two pairs of platinum wires were fixed in parallel in order to generate electric fields in multiple directions; this allows for the control of a BPM by *U*_*x*_, *U*_*y*_ on *x*-axis and *y*-axis. The control input voltages are determined autonomously by our algorithm. We set the maximum magnitude of the resultant voltage to be 20 V/cm. The chamber was filled with a motility buffer and 0.05% polyethyleneglycol (PEG).

Manual control of dynamic obstacles was implemented by applying magnetic fields. The strength of the applied magnetic field at the center of the chamber ranges from 1–5 mT; the strength of the field depends on the applied voltage. The velocity of the obstacle was varied depending on the strength of magnetic fields. The input voltage for moving obstacle ranges from 15–20 V. We tried to move obstacles to intercept a BPM by changing the direction of the magnetic field and the magnitude of the input voltage while the BPM approached the goal position in the experiment. The designed obstacles were not manipulated by dielectrophoretic force and it was confirmed through a simple experimental test.

A target BPM was traced based on a region detection method using image processing. Once the target BPM was distinguished on the image by tracking algorithm after setting an initial reference position for the BPM, the rest of binary image were used to calculate BD. All image processing and the computation of our algorithm were carried out with a sampling time of 0.16s. The stochastic model parameters *β*_*1*_, *β*_*2*_, *β*_*3*,_ and *β*_*4*_ were chosen after observing the motion of the BMP for 2–3 min and then used in the algorithm as constant values.

## Results

In experiments, different propertied BPMs were used and the input voltage for dynamic obstacles was changed during the experiments to generate different velocities.

### Single dynamic obstacle avoidance

In the first experiment, we demonstrated our suggested obstacle avoidance approach in the environment where there was one moving obstacle. The goal position was chosen manually and located at the opposite side across from the dynamic obstacle in the experiment (see S1 File). The weighting parameters *γ*, *δ*, and *σ* were 0.5 and *ω* of the *clearance* function was 0.7 in the objective function. In the case of the stochastic model, the parameters *β*_*1*_, *β*_*2*_, *β*_*3*_, and *β*_*4*_ were determined as ‒6.27, 1.83, 0.02 and 0.61, respectively using the trajectories of the BPM before running the algorithm. The safe range for the redefined *v*(*U*, *θ*) is 80 μm.

**Fig 8:**
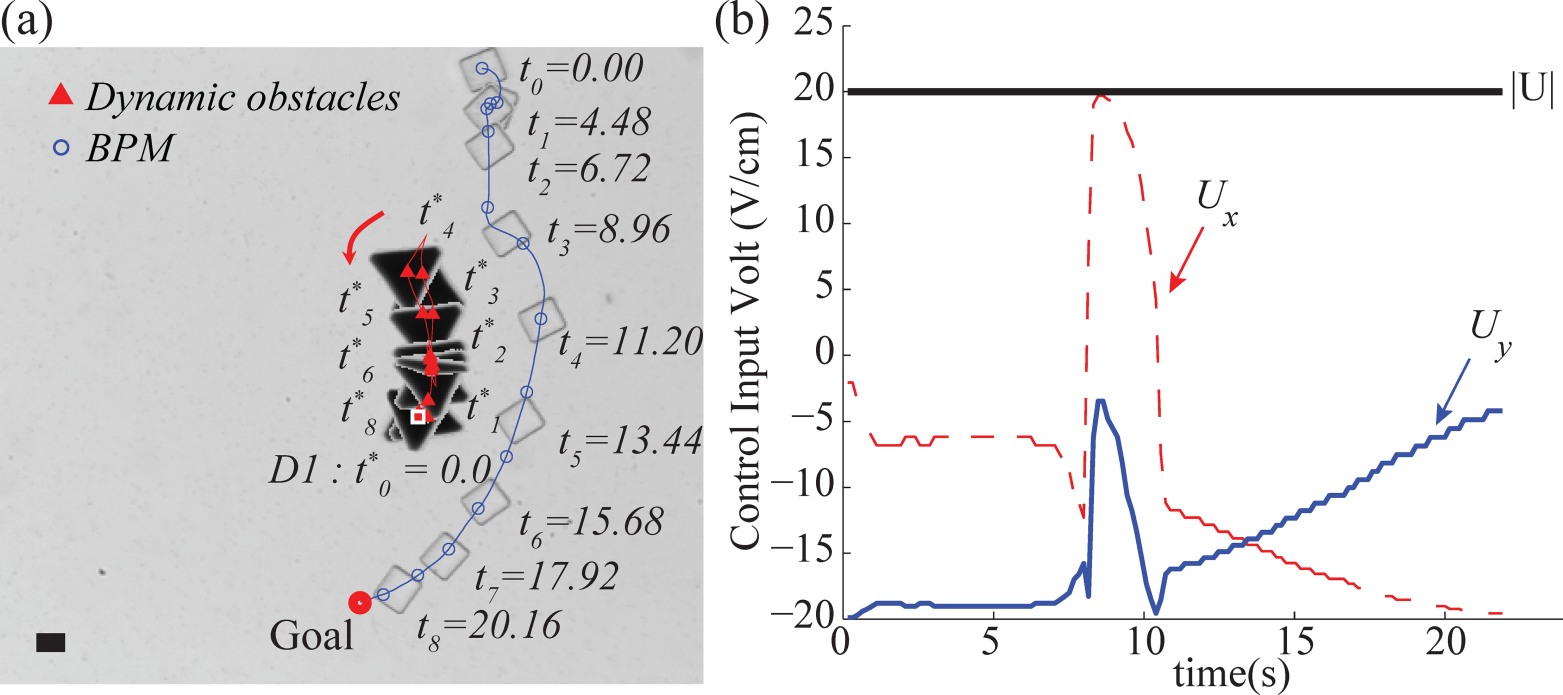
Experimental result of dynamic obstacle avoidance using a single obstacle. (a)Trajectories of the BPM and the dynamic obstacle (D1) (see also S1 File), (b)Control inputs on *x*-axis and *y*-axis in experiment. *U*_*x*_ represents voltage input on *x*-axis, *U*_*y*_ represents input voltage on *y*-axis, and |U| represents the magnitude of the resultant input voltage. The scale bar represents 20μm and *t*_*i*_, t^*^_i_ share the same time.

After obtaining all necessary parameters, the BPM was controlled by the proposed approach to reach the goal position without collision, as shown in Fig 8A. A single dynamic obstacle (D1) moves toward the BPM from the initial position at *t*_*o*_ (red triangle in Fig 8A). At *t* = 8.96s, the obstacle was moving closer to the BPM, as a result, the BPM was steered away from the obstacle by the computed control inputs. When the BPM passed the obstacle, we changed the moving direction of the obstacle at *t*_*4*_ = 11.20s and returned it to the initial position at *t*_*8*_ = 20.16s. Even though the obstacle was behind the BPM during *t*_*4*_—*t*_*8*_, the algorithm computed the control inputs that allowed the BPM to maintain a sufficient distance from the obstacle. To do so, the control inputs induced a wide curve trajectory to reduce the potential risk by the following obstacle. The determined control input was explained in Fig 8B. The magnitude of the resultant voltage was kept at the maximum value of 20 V/cm for the duration of the experiments.

**Fig 9:**
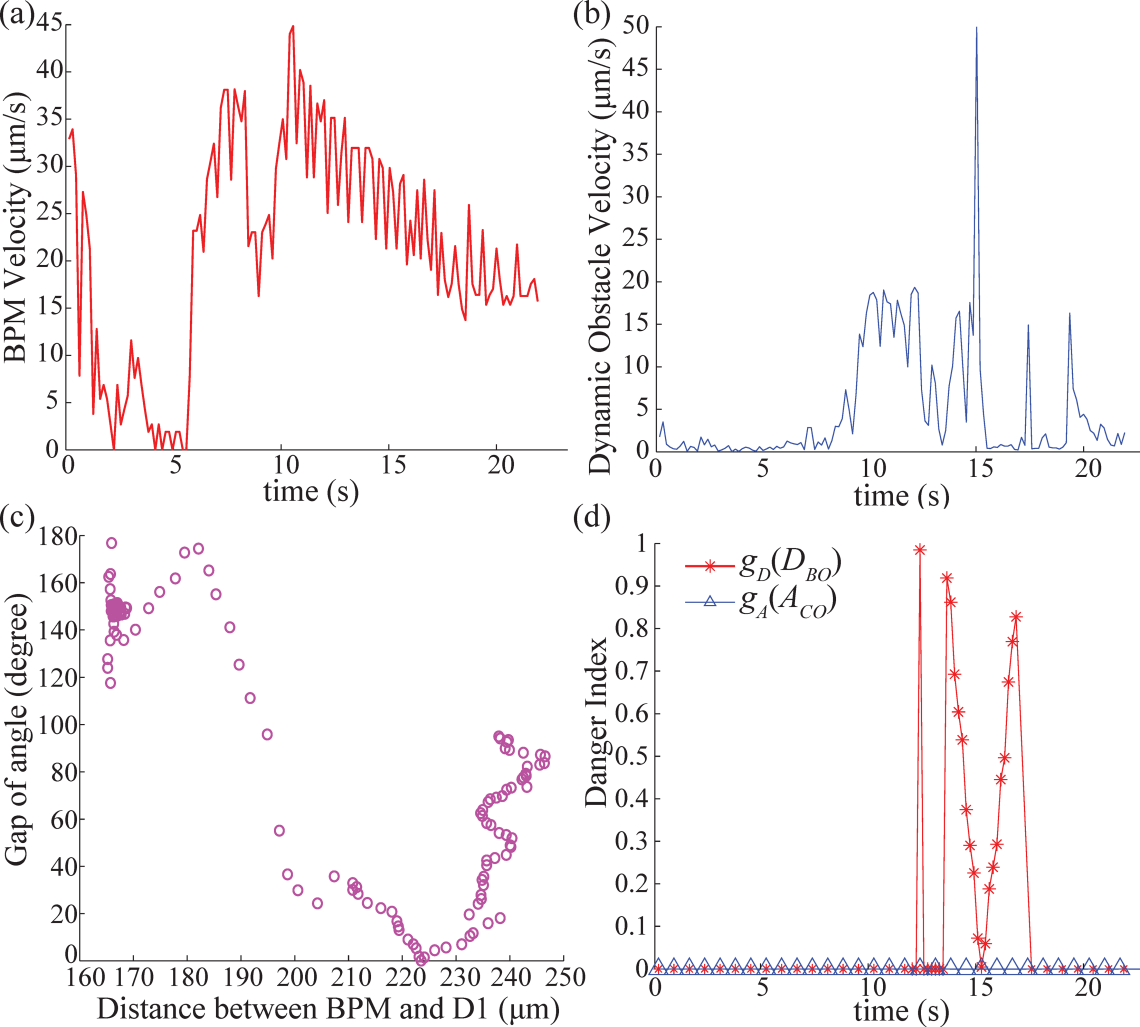
Analysis of experimental result data. (a)The velocity of the BPM, (b)The velocity of the dynamic obstacle (D1), (c)The control input depending on distance between the BPM and D1, with the gap between control input direction and the location angle of D1 from the BPM, (d)Danger index from the gap angle factor function *g*_*A*_(*A*_*CO*_) and the distance factor *g*_*D*_(*D*_*BO*_).

The velocities of BPM and the obstacle are described in Fig 9A and 9B. The average velocity of the BMP and the obstacle were 21.3 μm/s and 20 μm/s respectively. We steer the obstacle for interception when the BPM moved close to the obstacle.

The corresponding control input is plotted based on its angle gap from the heading angle towards D1 from the center of the BPM, and the distance between the BPM and D1 was indicated in Fig 9C. According to Fig 9C, the wide steering control was chosen when the distance between the BPM and D1 was within 190 μm. On the other hand, the control input had a small angle gap with the obstacle when the obstacle was outside of 200 μm distance from the BPM.

To analyze these characteristic of the control inputs with respect to the danger index, *g*_*A*_(*A*_*CO*_) and *g*_*D*_(*D*_*BO*_) were computed as shown in Fig 9D. In the case of *g*_*D*_(*D*_*BO*_), there are several peaks from 13s to 17s due to the high velocity of the obstacle. The value for *g*_*A*_(*A*_*CO*_), on the other hand, was 0 for the entire duration of the experiment. As a result, the product-based danger index (13) is also 0 for the entire experiment which means the corresponding control inputs did not have any potential risks.

### Avoidance motion under long term disturbance of obstacle

In this experiment, the proposed approach was implemented under longer following obstacle (see S2 File). All required parameters are shown in S1 Table (Exp2). The weight parameter *σ* had a relative high value compared with other weights. The weight parameters in the *movement* function have the smallest values. The safe range was 100 μm. The initial positions of the BPM and the dynamic obstacle (D1) were on the right side and the goal is at the left top.

**Fig 10:**
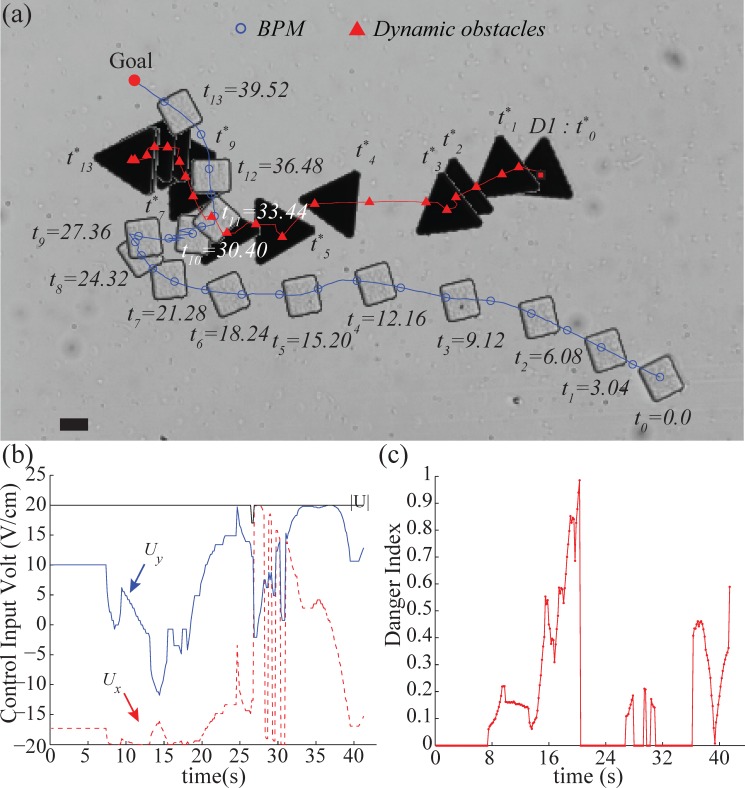
Experiment of avoiding obstacle following in parallel. (a)Trajectories of the BPM and dynamic obstacle (see also S2 File), (b)Control input voltages on *x*-axis and *y*-axis in experiment, (c)Resulting danger index using gap angle factor and distance factor, the scale bar represents 20 μm.

At the beginning, the BPM headed toward the goal with slight upward direction and D1 slowly moved left parallel with the BPM (Fig 10A). The velocity of D1 was increased to 57 μm/s at *t*_*3*_—*t*_*4*_. Then, we tried to make D1 intercept the BPM. The BPM maintained a distance with D1. When D1 stopped at *t*_*6*_, the BPM moved upward to approach the goal. At 27s, D1 was reactivated to intercept the BPM. Thus, the algorithm changes the control inputs to allow the BPM to detour and avoid collision at *t*_*9*_ (Fig 10A). The wide detour trajectory might be caused by the *control* function and the redefined VFH because the *control* function has high weight and tends to steer the BPM to empty space. In addition, the *v*(*U*,*θ*) function precludes the BPM from heading to the valley occupied by the obstacle. At the end, the BPM successfully reached the goal location without collision.

The BPM and the moving obstacle had the average velocities of 16 μm/s and 22 μm/s, respectively. The average distance maintained between the BPM and D1 is 30 μm under the applied control inputs, as shown in Fig 10B. In case of the danger index, the potential risk is computed as shown in Fig 10C. The danger index was low except for the moment when D1 stopped suddenly after it moved close to the BPM. However, after that moment, we can confirm that the algorithm performed safe control motion with lower danger index for 22–37s in Fig 10C. The average danger index is 0.15.

Compared with the previous experiment, the dynamic obstacle attempted to intercept the BPM in a manner which presented higher risk for collision. Nonetheless, the algorithm compensated for the higher risk and successfully guided the BMP to avoid the dynamic obstacle.

#### Avoidance motion using strong self-actuated BPMs

To demonstrate the controllability of our algorithm, we conducted several experiments using BPMs with strong self-actuated motion which increase uncertainties in motion control (see S3 File).

The column of ‘Exp3’ in S1 Table indicates all parameters for the algorithm with a safe range of 100 μm. The BPM had a large *β*_*3*_ value which enables the BPM to have a fast angular velocity. Also, the parameters relative to translational motion are large comparing with other cases.

**Fig 11:**
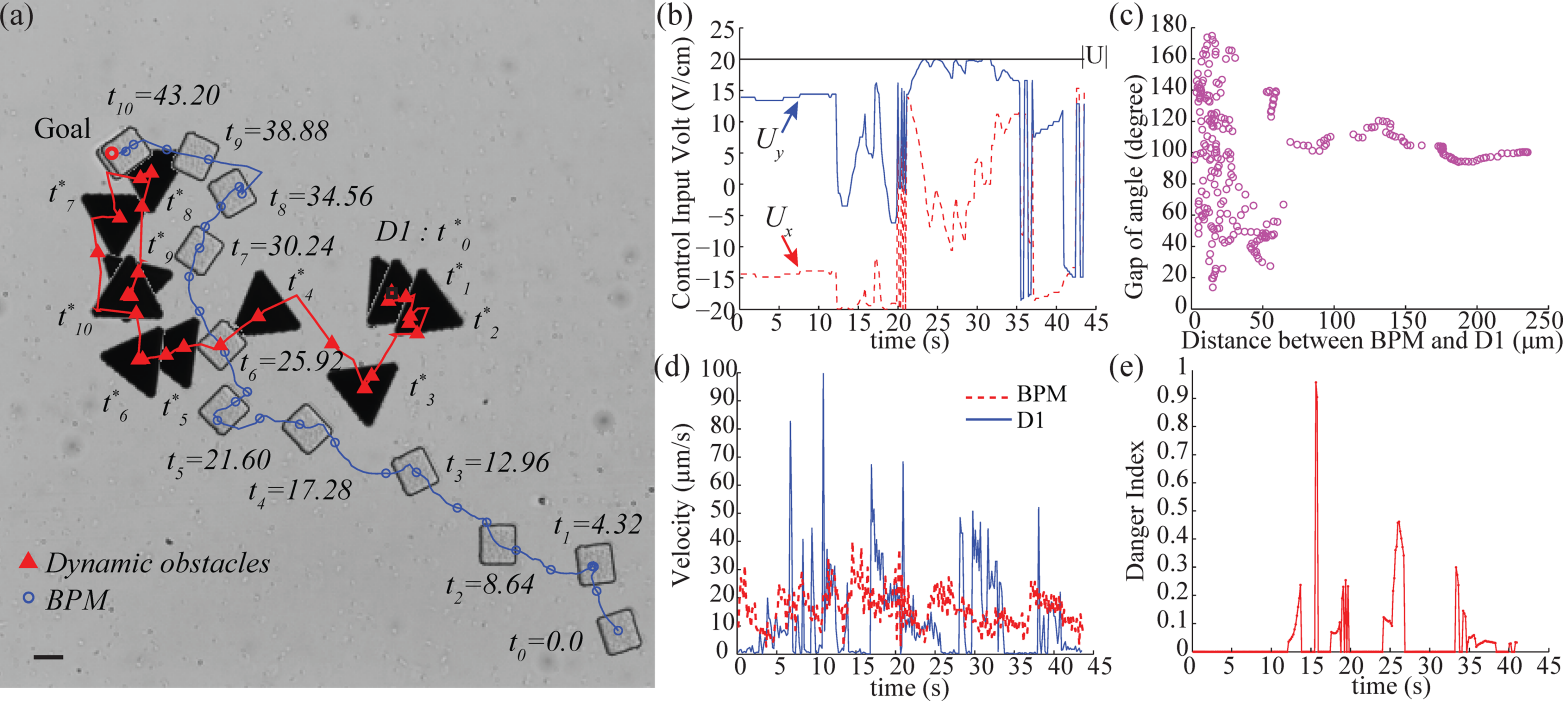
Experiment of using strong self-actuation BPM. (a)Trajectories of the BPM and dynamic obstacle (see also S3 File), (b)The control input voltages in experiment, (c)The control input depending on distance between the BPM and D1, with the gap between control input direction and the location angle of D1 from the BPM, (d)Velocities of the BPM and the dynamic obstacle, (e)Resulting danger index using gap angle factor and distance factor, the scale bar is 20 μm.

The trajectories of the BPM and the dynamic obstacle (D1) are present in Fig 11A. The BPM started at the right bottom corner and the goal was at the left top corner. During 0–10s, the BPM and the D1 were position far apart. After 10s, the potential risk increased due to closing distance between D1 and the BPM. As the distance becomes smaller at 15s, the strong self-actuated motion began to lead to a higher potential risk compared with the previous experiments, as shown in Fig 11A and 11E. However, the BPM avoided collision due to the control inputs computed using low danger index. While D1 stayed in front of the BPM, the BPM was able to continuously dodge the moving obstacle in real-time. Notably, the BPM was waiting at the right side of the goal area while D1 was occupying the goal location. Once D1 left from the goal location, the BPM went to the goal position from the place where it waited; much like a midfielder passing the defenders to score a goal. All the control inputs are depicted in Fig 11B. Regarding the distance between the BPM and D1 in Fig 11C, the chosen control inputs ranged in the avoiding angle from 19° to 180° and populate around 100°. Even though there was enough distance such as 100–250 μm between the BPM and D1, the control input gave wide spatial motion with the obstacle considering the strong self-actuation in terms of angle.

The velocity of the BPM and the dynamic obstacle are shown in Fig 11D. Running the obstacle avoidance algorithm, the BPM kept average distances of 62 μm from the obstacle. Due to the time-varying velocity of the obstacle, the given control input induces low values of danger index as explained in Fig 11E. There is no potential risk most of times with an average danger index of 0.23.

Through this experiment, the capability of our suggested approach was examined and verified. This demonstrated safe motion planning of self-actuated BPM in the presence of an aggressive dynamic obstacle.

### Multiple dynamic obstacle avoidance

So far, our experiments demonstrated dynamic obstacle avoidance using single dynamic obstacle. To ensure that our method is usable with multiple dynamic obstacles, we carried out two experiments, each using two identical dynamic obstacles. Due to the use of a global magnetic field and the motion of the two identical obstacles will be the same. The all parameters are indicated on S1 Table.

**Fig 12:**
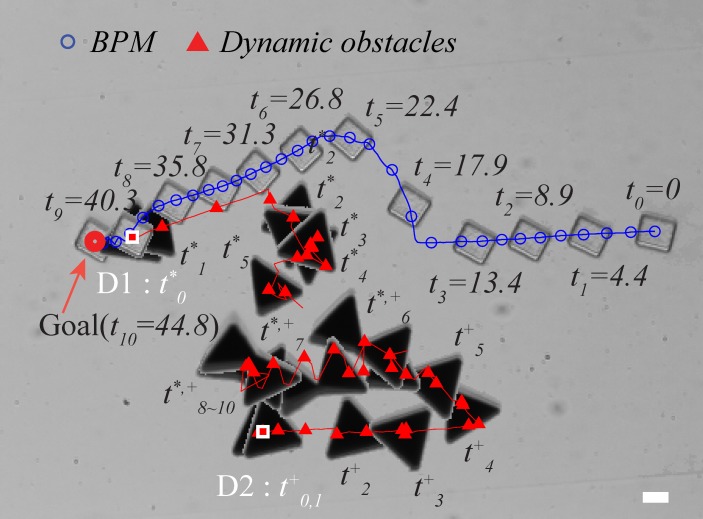
Experiment of using multiple obstacles. (see also S4 File). The scale bar represents 20 μm.

The first experiment (Exp4) started with two obstacles positioned at the midpoint between the BPM and the goal location (see S4 File). Fig 12 illustrates the resultant trajectories of the BPM and the two obstacles (D1, D2). Up until 16s, the BPM was moving closer to D1 and D2 as described in Fig 12. When the BPM encountered the nearest obstacle D1, the derived control input avoided the valley placed between the D1 and D2 because the valley was regarded as a dangerous area by *clearance* function and *v*(*U*,*θ*). Thus, the BPM headed to the upper path and got out of the valley as depicted in Fig 12. We tried to move D1 and D2 backward in order to intercept the BPM again. However, D1 and D2 bonded together due to magnetic force. After their bonding, they moved together as one larger obstacle.

**Fig 13:**
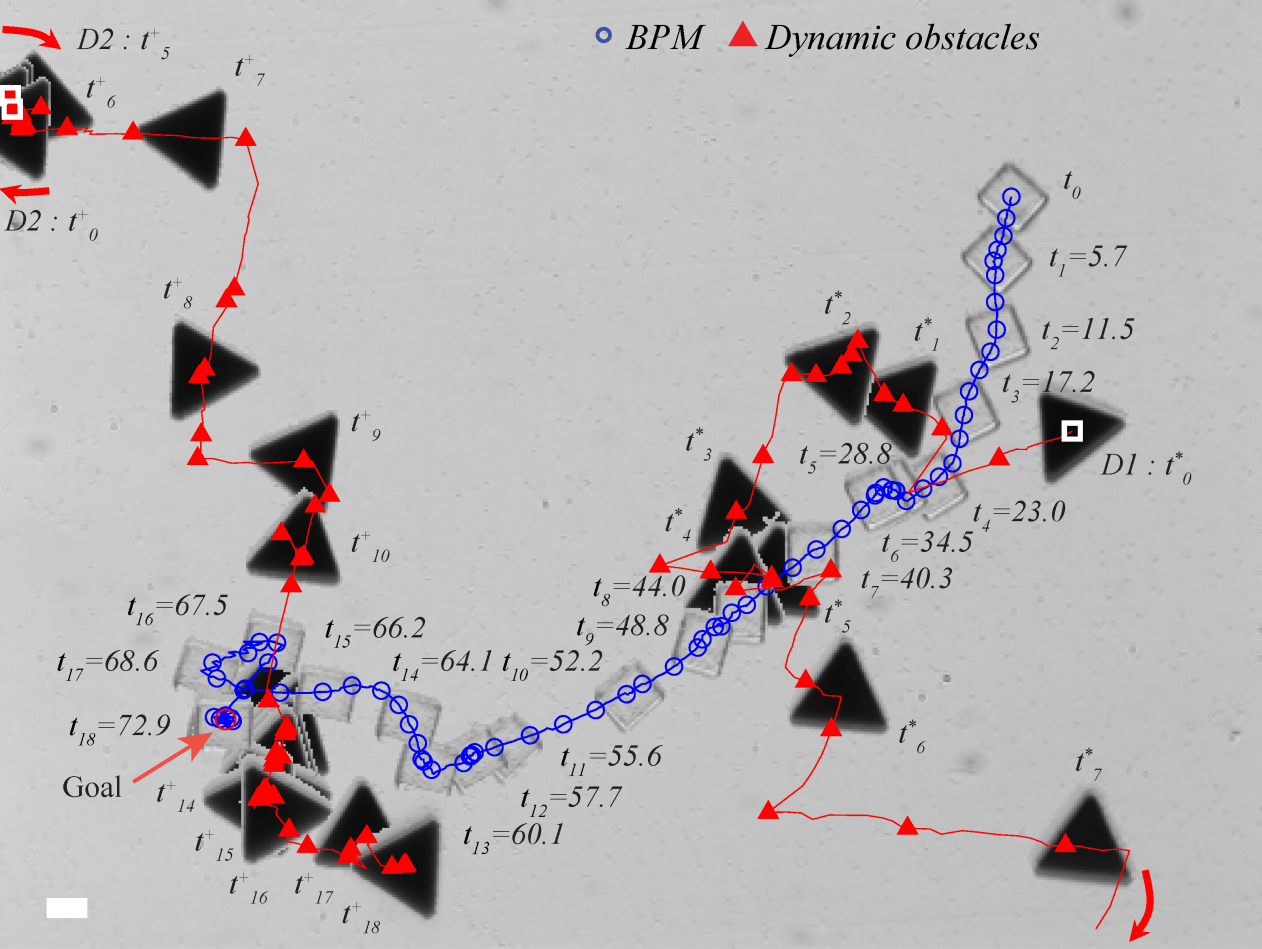
Experiment of using multiple obstacles. The scale bar represents 20 μm.

In the second case, two obstacles were initially located at separate places. D1 intercepted the BPM’s motion from the bottom side at *t*_*1*_ and *t*_*3*_ as illustrated in Fig 13. After the BPM cleared D1 by moving downward, D2 came down from the top to attempt an interception. After 52s (*t*_*8*_), D2 was within the range to present a potential risk. The BPM could not approach the goal location because the D2 occupied the goal area. When D2 moved away from the goal, the BPM was rotating around the region at *t*_10_—*t*_*12*_ and eventually reach the goal position.

**Fig 14:**
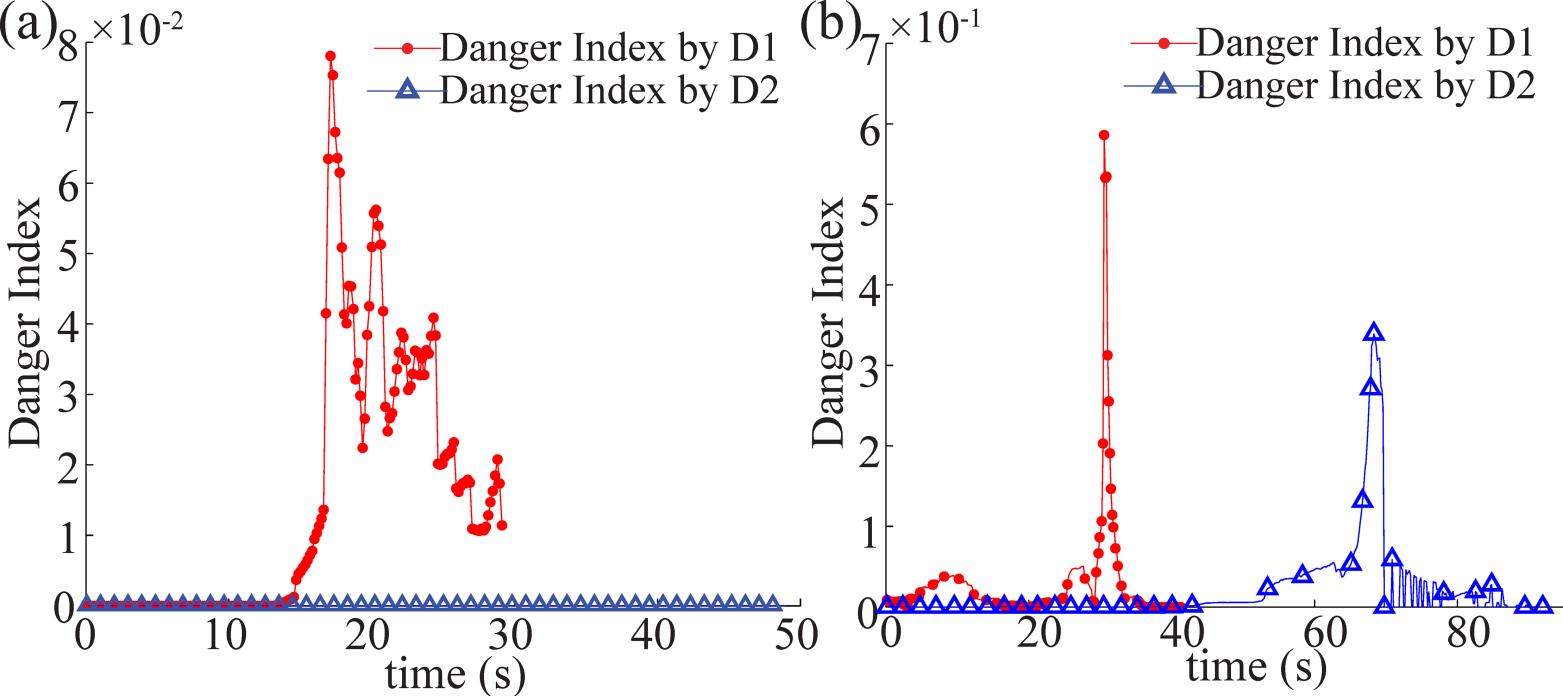
Computation of danger index. (a)Resulting danger index using the gap angle factor and the distance factor with D1, D2 in Fig 12, (b)Resulting danger index using the gap angle factor and the distance factor with D1,D2 in Fig 13.

The danger indexes for each obstacle were calculated (Fig 14). Fig 14A shows the danger index of the first case. There was low a potential risk due to the wide detour path planning to avoid the valley. Similarly, in the second case, the danger index was below 0.7 while the BPM avoided D1 and D2 (Fig 14B). Comparing to the first case, the second case has greater danger index.

In this experiment, we show that our autonomous motion strategy can guide a BPM to avoid the interference from multiple dynamic obstacles and compute a safe trajectory to the target position.

## Conclusions

The dynamic obstacle avoidance approach was demonstrated by successfully guiding BPMs to avoid multiple nickel coated dynamic obstacles. To successfully implement the algorithm in experiments, we took into account the uncontrollable motion of BPMs and the distorted electric field near the obstacles. Our suggested objective function includes four functions for extracting optimal control inputs. The sub functions of the objective function are *heading*, *movement*, *clearance*, and *control*. The algorithm determines the safe motion control input that maximizes four performance categories—goal arrival, no collision, speed, and controllability. The exerted control inputs are selected from the admissible control input window during a sampling time. Instead of adding other functions to the objective function, the redefined VFH concept is utilized to eliminate control inputs which may lead to collision.

Five experiments have been executed in different setup with various circumstances. Moreover, we used strong self-actuated BPMs in experiments in order to evaluate the robustness of the control system. Our proposed approach allows the target BPMs to arrive at the goal positions without collision with single or multiple dynamic obstacles. To evaluate the performance of each result motion, the danger index was applied to compare the potential risk caused by the chosen control input. The danger index is computed using two factors that are how much the control input can steer the BPM from the obstacle and what is the distance between the BPM and obstacles. Through danger-evaluation method, we can quantify the performance of the results in terms of safety. All experiments have low average danger index values.

We believe the successful implementation of the dynamics obstacle avoidance algorithm demonstrated the viability for using autonomous systems to control microrobots for accurate navigation. For future work, the dynamic obstacle algorithm will be implemented in an environment with unpredictable moving obstacles.

## Supporting information

S1 Table Parameters for experiments. (PDF)

S1 File Video of dynamic obstacle avoidance Exp1. (MP4)

S2 File Video of dynamic obstacle avoidance Exp2. (MP4)

S3 File Video of dynamic obstacle avoidance Exp3. (MP4)

S4 File Video of multi dynamic obstacle avoidance Exp4. (MP4)

S5 File Supporting information for fabrication of BPM. (PDF)

## References

[1] Abbott JJ, Nagy Z, Beyeler F, Nelson BJ. Robotics in the small, part I: microbotics. IEEE Rob Autom Mag. 2007;14(2):92–103.

[2] Sakar MS, Steager EB, Julius AA, Kim M, Kumar V, Pappas GJ, editors. Biosensing and actuation for microbiorobots. Robotics and Automation (ICRA), 2010 IEEE International Conference on; 2010: IEEE.

[3] Hassan U, Bajaj P, Damhorst G, Bashir R, editors. Biomedical micro and nanotechnology: From lab-on-chip to building systems with cells. Solid-State Sensors, Actuators and Microsystems, 2013 Transducers & Euroscensors 17th, International Conference on; 2013: IEEE.

[4] Martel S, editor Targeted delivery of therapeutic agents with controlled bacterial carriers in the human blood vessels. 2006 Bio Micro and Nanosystems Conference; 2006.

[5] Jeong S, Choi H, Go G, Lee C, Lim KS, Sim DS, et al Penetration of an artificial arterial thromboembolism in a live animal using an intravascular therapeutic microrobot system. Med Eng Phys. 2016. 10.1016/j.medengphy.2016.01.001 26857290

[6] Li H, Go G, Ko SY, Park J-O, Park S. Magnetic actuated pH-responsive hydrogel-based soft micro-robot for targeted drug delivery. Smart Mater Struct. 2016;25(2):027001.

[7] Dauge M, Gauthier M, Piat E. Modelling of a planar magnetic micropusher for biological cell manipulations. Sens Actuat A. 2007;138(1):239–47.

[8] Steager EB, Sakar MS, Magee C, Kennedy M, Cowley A, Kumar V. Automated biomanipulation of single cells using magnetic microrobots. Int J Rob Res. 2013;32(3):346–59.

[9] Pieters R, Tung H-W, Charreyron S, Sargent DF, Nelson BJ, editors. RodBot: A rolling microrobot for micromanipulation. Robotics and Automation (ICRA), 2015 IEEE International Conference on; 2015: IEEE.

[10] Purcell EM. Life at low Reynolds number. Am J Phys. 1977;45(1):3–11.

[11] Zhang L, Abbott JJ, Dong L, Kratochvil BE, Bell D, Nelson BJ. Artificial bacterial flagella: Fabrication and magnetic control. Appl Phys Lett. 2009;94(6):064107–10.

[12] Behkam B, Sitti M. Bacteria integrated swimming microrobots. 50 years of artificial intelligence. 4850: Springer; 2007. p. 154–63.

[13] Di Leonardo R, Angelani L, Dell’Arciprete D, Ruocco G, Iebba V, Schippa S, et al Bacterial ratchet motors. Proc Natl Acad Sci USA. 2010;107(21):9541–5. doi: 10.1073/pnas.0910426107 20457936 10.1073/pnas.0910426107 PMC2906854

[14] Julius AA, Sakar MS, Steager E, Cheang UK, Kim M, Kumar V, et al., editors. Harnessing bacterial power in microscale actuation. Robotics and Automation (ICRA), 2009 IEEE International Conference on; 2009: IEEE.

[15] Honda T, Arai K, Ishiyama K. Micro swimming mechanisms propelled by external magnetic fields. IEEE Trans Magn. 1996;32(5):5085–7.

[16] Diller E, Giltinan J, Sitti M. Independent control of multiple magnetic microrobots in three dimensions. Int J Rob Res. 2013;32(5):614–31.

[17] Parmar J, Ma X, Katuri J, Simmchen J, Stanton MM, Trichet-Paredes C, et al Nano and micro architectures for self-propelled motors. Sci Technol Adv Mater. 2016. 10.1088/1468-6996/16/1/014802 PMC5036491 27877745

[18] Yesin KB, Vollmers K, Nelson BJ. Modeling and control of untethered biomicrorobots in a fluidic environment using electromagnetic fields. Int J Rob Res. 2006;25(5–6):527–36.

[19] Kim D, Brigandi S, Julius AA, Kim MJ, editors. Real-time feedback control using artificial magnetotaxis with rapidly-exploring random tree (RRT) for Tetrahymena pyriformis as a microbiorobot. Robotics and Automation (ICRA), 2011 IEEE International Conference on; 2011 9–13 May 2011.

[20] Charreyron S, Pieters RS, Hsi-Wen T, Gonzenbach M, Nelson BJ, editors. Navigation of a rolling microrobot in cluttered environments for automated crystal harvesting. Intelligent Robots and Systems (IROS), 2015 IEEE/RSJ International Conference on; 2015 Sept. 28 2015-Oct. 2 2015.

[21] Kim H, Cheang UK, Kim MJ, Lee K, editors. Obstacle avoidance method for microbiorobots using electric field control. Cyber Technology in Automation, Control, and Intelligent Systems (CYBER), 2014 IEEE 4th Annual International Conference on; 2014 4–7 June 2014.

[22] Kim H, Kim MJ. Electric Field Control of Bacteria-Powered Microrobots Using a Static Obstacle Avoidance Algorithm. IEEE Trans Rob. 2016;32(1):125–37. doi: 10.1109/TRO.2015.2504370

[23] Steager EB, Sakar MS, Kim DH, Kumar V, Pappas GJ, Kim MJ. Electrokinetic and optical control of bacterial microrobots. JMicromech Microeng. 2011;21(3):035001.

[24] Kim H, Cheang UK, Julius AA, Min Jun K, editors. Dynamic obstacle avoidance for bacteria-powered microrobots. Intelligent Robots and Systems (IROS), 2015 IEEE/RSJ International Conference on; 2015 Sept. 28 2015-Oct. 2 2015.

[25] Copeland MF, Weibel DB. Bacterial swarming: a model system for studying dynamic self-assembly. Soft Matter. 2009;5(6):1174–87. doi: 10.1039/B812146J 23926448 10.1039/B812146J PMC3733279

[26] Murray P, Rosenthal K, Kobayashi G, Pfaller M. Enterobacteriaceae. St.Louis, MO1994. 227–34 p.

[27] Steager E, Kim C-B, Patel J, Bith S, Naik C, Reber L, et al Control of microfabricated structures powered by flagellated bacteria using phototaxis. Applied Physics Letters. 2007;90(26):263901.

[28] Linder V, Gates BD, Ryan D, Parviz BA, Whitesides GM. Water‐Soluble Sacrificial Layers for Surface Micromachining. small. 2005;1(7):730–6. doi: 10.1002/smll.200400159 17193516 10.1002/smll.200400159

[29] Kim H, Cheang UK, Kim D, Ali J, Kim MJ. Hydrodynamics of a self-actuated bacterial carpet using microscale particle image velocimetry. Biomicrofluidics. 2015;9(2):024121 doi: 10.1063/1.4918978 26015833 10.1063/1.4918978 PMC4409625

[30] Wong D, Beattie EE, Steager EB, Kumar V. Effect of surface interactions and geometry on the motion of micro bio robots. Appl Phys Lett. 2013;103(15):153707.

[31] Berg HC, Anderson RA. Bacteria swim by rotating their flagellar filaments. Nature. 1973;245:380–2. 4593496 10.1038/245380a0

[32] Fox D, Burgard W, Thrun S. The dynamic window approach to collision avoidance. IEEE Rob Autom Mag. 1997;4(1):23–33.

[33] Borenstein J, Koren Y. The vector field histogram-fast obstacle avoidance for mobile robots. IEEE Trans Rob. 1991;7(3):278–88.

