# Supplementary material for: Autonomous dynamic obstacle avoidance for bacteria-powered microrobots (BPMs) with modified vector field histogram
Source: PLoS One. 2017 Oct 11;12(10):e0185744. doi: 10.1371/journal.pone.0185744 (PMC5636095; doi:10.1371/journal.pone.0185744)
Supplement: S1 Table — (PDF) [file pone.0185744.s001.pdf]

S1. Table. Parameters for experiments

Parameter	Exp1	Exp2	Exp3	Exp4	Exp5
γ	0.5	0.3	0.3	0.3	0.3
δ	0.5	0.2	0.2	0.4	0.2
ω	0.5	0.5	0.5	0.6	0.7
σ	0.5	0.7	0.6	0.5	0.5
β_1	-6.27	1.03	-7.65	-1.89	5.01
β_2	1.83	-2.23	-12.59	5.03	6.20
β_3	0.02	-0.26	7.11	0.01	0.08
β_4	0.61	0.92	0.69	0.76	0.81
