# Supplementary material for: Autonomous dynamic obstacle avoidance for bacteria-powered microrobots (BPMs) with modified vector field histogram
Source: PLoS One. 2017 Oct 11;12(10):e0185744. doi: 10.1371/journal.pone.0185744 (PMC5636095; doi:10.1371/journal.pone.0185744)
Supplement: S5 File — (PDF) [file pone.0185744.s006.pdf]

Processing of manufacturing Bacteria-Powered Microrobot

1. Bacteria culture

Agar plate

1) LB plate 0.6% Agar plate

➔Bacto tryptone (Difco): 10g

➔Yeast Extract: 5g

➔NaCl: 5g

➔Bacto Agar (Difco): 6g

2) Dissolve the above into 1000 ml H_2O (Ultrapure H_2O).

3) Autoclave in 2000 ml Flask.

4) Pour 10 × 100 ml into sterile bottles @ 100 ml/bottle.

5) Store at room temperature.

6) Microwave the 100 ml bottle on very low for 10 min.

7) Then add sterile glucose: 2 mls of a 25% solution/100ml of agar

8) Pour plates:

➔ Large plates (for Serratia marcescens) are 150 × 15 mm

(Cat. No. 25384-326 from VWR)

25 ml / plate

➔ Smaller (standard) are 100 × 15 mm

15 ml/plate

9) Then let's cool the plate.

2. Microfabrication

1) Dextran Sacrificial Layer

Prepare 5% (w/v) dextran aqueous solution (dextran around 66 kDa molecular weight)

- This may range from 2.5-20%

Solution may be filtered in 0.45 μm filters

Dispense on clean substrate.

Spin coat at 3000 rpm ramping slowly

- This may range from 1000-4000 rpm

Bake at 95-150º C for 2 minutes to remove water.

- 125º C seems to work well

2) Pretreatment

Acetone/Isopropanol Rinse

DI Rinse

Dehydrate 200ºC for 5 minutes

Mark top of substrate if clear to tell orientation for handling

3)Coat (3 µm)

Static dispense 1 ml per inch of diameter

Spread cycle: Ramp to 500 rpm @ 100 rmp/sec, hold for 5-10 seconds, ramp to final spin speed of 2000 rpm @ 300 rpm/sec, hold for 30 seconds

4)Soft Bake

Pre-bake 1 minute @ 65ºC

Soft-bake 3 minutes @ 95ºC

5)Expose

Total energy dose is 75-100 mJ/cm^2

6)Post Exposure Bake

1 minute @ 65ºC

1 minute @ 95ºC

7)Develop

Immersion develop for 1 minute in SU-8 Developer

8)Rinse & Dry

3. Blotting for attaching bacteria to microstructure

1)Cut the glass that has pattern of microstructures.

2)Put the piece of pattern on the edge of colony at agar plate after facing the pattern toward the agar plate.

3)Gently rinse inside of 5 µl tube that has DI water.

4)Transfer the volume of DI water from the 5 µl tube to the experimental chamber.

The information can also be found **DOI 10.1109**.
